# Effects of *Rab7* gene up-regulation on renal fibrosis induced by unilateral ureteral obstruction

**DOI:** 10.1590/1414-431X20209220

**Published:** 2020-04-06

**Authors:** Qing Xu, Lei Liu, Yiqiong Yang, Zhi Wang, Yingying Cai, Tingting Hong, Pingsheng Chen

**Affiliations:** 1 Department of Pathology, Affiliated Hospital of Jiangnan University, Wuxi, Jiangsu, China; 2 Department of Pathology, Zhongshan Hospital, Fudan University, Shanghai, China; 3 Department of Pathology and Pathophysiology, School of Medicine, Southeast University, Nanjing, Jiangsu, China; 4 Department of Oncology, Affiliated Hospital of Jiangnan University, Wuxi, Jiangsu, China

**Keywords:** Rab7, Autophagy, Renal fibrosis, Crispr/cas9, Endocytosis, Lipophagy

## Abstract

Rab7, an important member of the Rab family, is closely related to autophagy, endocytosis, apoptosis, and tumor suppression but few studies have described its association with renal fibrosis. In the early stage, our group studied the effects of *Rab7* on production and degradation of extracellular matrix in hypoxic renal tubular epithelial cells. Because cell culture *in vitro* is different from the environment *in vivo*, it is urgent to understand the effects *in vivo*. In our current study, we established a renal fibrosis model in *Rab7*-knock-in mice (prepared by CRISPR/Cas9 technology) and wild type (WT) C57BL/6 mice using unilateral ureteral obstruction (UUO). Seven and 14 days after UUO, the expression of the Rab7 protein in WT mice, as well as the autophagic activity, renal function, and the degree of renal fibrosis in WT and *Rab7*-knock-in mice were examined by blood biochemical assay, hematoxylin-eosin and Masson staining, immunohistochemistry, and western blotting. We found that the *Rab7* expression in WT mice increased over time. Furthermore, the autophagic activity constantly increased in both groups, although it was higher in the *Rab7*-knock-in mice than in the WT mice at the same time point. Seven days after UUO, the degree of renal fibrosis was milder in the *Rab7*-knock-in mice than in the WT mice, but it became more severe 14 days after surgery. Similar results were found for renal function. Therefore, *Rab7* suppressed renal fibrosis in mice initially, but eventually it aggravated fibrosis with the activation of autophagy.

## Introduction

The Rab family of proteins is a member of the Ras superfamily and exists in almost all mammals. Rab7, the most studied member of the Rab family, has been found to play a crucial role in vesicle transport in cells (1). Rab7 is involved in the transport from early endosomes to late endosomes, and later in the trafficking from endocytic vesicles to lysosomes. Moreover, it is closely associated with autophagy, endocytosis, apoptosis, tumor suppression, and even neurons (2–6). *Rab7* dysfunction is associated with a wide range of human diseases (7–10), however, few studies have addressed the relationship between *Rab7* and renal interstitial fibrosis. It has been shown that *Rab7* is involved in the formation and transport of autophagosomes and their subsequent binding to lysosomes (3 [Bibr B04] [Bibr B05],^11^). Autophagy has been shown to reduce extracellular matrix deposition and renal tubule atrophy, thereby alleviating the degree of renal fibrosis (12–16). Using *LC3B*-knockout mice, Ding et al. (14 [Bibr B15]) demonstrated that autophagy inhibited TGF-β expression and played a protective role in avoiding excessive accumulation of collagen fibers outside the cell. In contrast, other studies have suggested that autophagy can aggravate renal fibrosis. Using the Atg7-knockout mouse model and the autophagy inhibitors CQ and 3-MA, Livingston et al. (17) found that renal fibrosis was alleviated after autophagy was inhibited. Furthermore, using CQ and 3-MA, Yan et al. (18) suggested that the activation of autophagy continuously promotes lipid deposition. Thus, the exact effect of *Rab7* expression on renal fibrosis is not yet known based on its effect of autophagic activity.

The prevalence of chronic kidney disease has risen in China annually, and renal fibrosis is an inevitable process that ultimately leads to end-stage renal failure (19,20). Therefore, the search for new therapeutic targets that can suppress or delay renal fibrosis is important for improving the quality of life of patients with chronic kidney disease and for reducing the case fatality rate.

Previously, our group had studied the relationship between autophagy and renal fibrosis only *in vitro* and found that the expression of Col-IV was significantly increased when HK-2 cells were cultured under hypoxia and 3-MA (21). In the current study, we established a renal fibrosis model in *Rab7*-knock-in mice (prepared by CRISPR/Cas9 technology) (22) and wildtype (WT) C57BL/6 mice using unilateral ureteral obstruction (UUO). *Rab7* expression in WT mice, as well as the autophagic activity, renal function, and renal fibrosis in different mouse groups were observed at different time points after operation to investigate the relationship between *Rab7* and renal fibrosis, and to explore its possible mechanism, and thus pave the way for further studies on the role of *Rab7* as a new therapeutic target for renal fibrosis.

## Material and Methods

### Animals

This study was approved by the Animal Research Ethics Committee of the School of Medicine, Southeast University. Five heterozygous *Rab7*-knock-in mice (three males and two females) were provided by Cyagen (China). Using the latest CRISPR/Cas9 genome editing technique, gRNA was specifically introduced into the ROSA26 locus of C57BL/6 mice. Together with a plasmid containing mouse *Rab7* cDNA and *cas9*, the gRNA was injected into fertilized mouse eggs by prokaryotic microinjection to establish *Rab7*-knock-in mice. The *Rab7* homozygous mice needed for this study were obtained through breeding of these transgenic mice. C57BL/6 mice were purchased from Maijiesi (China). All the mice were fed by water and ordinary feed. The mice were kept at the Laboratory Animal Center, School of Medicine, Southeast University in cages in a room at a constant temperature of 23°C and a constant humidity of 55±5%.

### Experimental design

Twenty WT C57BL/6 mice and 20 homozygous mice were divided into two groups, with 10 WT C57BL/6 mice and 10 homozygous mice in each group. In each group, four mice were in the sham-operation group and six were in the UUO group. Seven and 14 days after the UUO, mice were sedated by 4% chloral hydrate (350 mg/kg) that was injected into the abdominal cavity, the kidneys were then harvested for hematoxylin-eosin (HE) and Masson staining, immunohistochemistry, and western blot analysis, and after which the mice were euthanized.

### UUO mouse model

Mice that underwent UUO treatment were aged 8 weeks (±2 days), with a body weight of 25±3 g. Mice were anesthetized by intraperitoneal administration of 4% chloral hydrate (350 mg/kg). One side of the mouse was then fixed on the operating table, the body hair was removed, and the operating area was disinfected with iodophor. An incision was made in the back near the spine. The skin, muscles, and abdominal wall were dissected layer by layer to expose and dissociate the ureter on one side. The ureter was ligated with a 4-0 suture near the inferior pole of the kidney, and then at the distal side. The incision was sutured layer-by-layer to end the procedure. In the sham-operation group, only one side of the ureter was exposed, which was then directly sutured layer by layer, without any treatment.

### Blood biochemical assay

Blood samples were taken from the hearts of mice when sedated by 4% chloral hydrate (350 mg/kg). The samples were analyzed for serum creatinine (Scr) and urea nitrogen (BUN) using colorimetric kits (BioAssay Systems, USA).

### DNA extraction and agarose gel electrophoresis

Direct Mouse Genoytping Kit (APExBIO, USA) was used to extract DNA directly from the mouse tail, and DNA samples were amplified into cDNA with primers by PCR. The thermal cycling conditions were as follows: predenaturation for 3 min at 94°C followed by the amplification reaction consisting of 33 cycles of denaturation for 30 s at 94°C, annealing for 35 s at 60°C, extension for 35 s at 72°C, and eventual extension for 5 s at 72 min. The following primers of *Rab7* were used: 5′-CACTTGCTCTCCCAAAGTCGCTC-3′; 5′-ATACTCCGAGGCGGATCACAA-3′; 5′-AGATGTACTGCCAAGTAGGAAAGTC-3′. The 1.5% agarose solution was prepared with 1× TAE solution (Beyotime, China) and agarose (Sigma, USA). The cDNA samples were resolved by electrophoresis and stained with SuperRed/GelRed (Biosharp, China) in the agarose solution at a ratio of 0.01%. The DNA bands were observed by an ultraviolet transmission detector (Jingke, China) at the end of electrophoresis.

### RNA isolation and quantitative real-time PCR

The Trizol method (Invitrogen, USA) was used to extract RNA from the mouse tail. Subsequently, HiScript II Q RT SuperMix for qPCR (+gDNA wiper) (Vazyme, China) was used to reverse-transcribe the RNA. Quantitative real-time PCR (qRT-PCR) was performed using the ChamQ Universal SYBR qPCR Master Mix (Vazyme) in a Step One Plus real-time PCR system (Applied Biosystems, USA). The following primers were used: *Rab7* forward 5′-TGATGGTGGACGACAGACTTG-3′ and reverse 5′-GCTGGCCTGGATGAGAAACTC-3′; β-actin forward 5′-AAGTGTGACGTTGACATCCGTAAA-3′ and reverse 5′-CAGCTCAGTAACAGTCCGCCTAGA-3′. The gene expression level of β-actin served as the control of target genes for reaction efficiency. Gene expression was quantitatively analyzed using the 2^-ΔΔCT^ method.

### Protein extraction and western blotting

Fresh kidney samples from each group were ground with grinding rods and lysed with RIPA buffer (Beyotime, China) containing PMSF and protease inhibitors (APExBIO). After incubation on ice for 30 min, the supernatant was obtained by centrifugation at 12,000 *g* for 10 min at 4°C. The protein concentration was determined by the BCA method. Then, the samples were loaded onto a gel for electrophoresis. The proteins in the gel were then transferred to a PVDF membrane, which was blocked with 5% skim milk in TBST at 37°C for 1 h, followed by incubation at room temperature with Col-I, α-SMA, TGF-β, Rab7, LC3B, Beclin-1, or GAPDH primary antibodies for 90 min. The membrane was washed twice with TBST for 15 min, and then incubated with goat anti-rabbit secondary antibodies at room temperature for 1 h. After the membrane was washed twice with TBST for 15 min, the blots were developed with ECL (Vazyme), and the results were analyzed with ImageJ software (Media Cybernetics Inc., USA).

### Histopathology

The kidneys of mice from the different groups were harvested, fixed in 4% paraformaldehyde, embedded in paraffin, and then sectioned. The structural integrity of the kidneys was observed by HE staining, and the deposition of interstitial collagen fibers was observed by Masson staining to assess the degree of renal fibrosis.

### Immunohistochemistry

Kidney sections of each group were incubated overnight at 4°C with Rab7, Col-I, α-SMA, or TGF-β primary antibodies, and then with goat anti-rabbit secondary antibodies at 37°C for 20 min. The sections were then developed with DAB. The sections were observed and photographed under a light microscope (×200, Zeiss, Germany). The positive areas were quantitatively analyzed using the ImageJ software.

### Statistical analysis

Results are reported as means±SE. One-way ANOVA with Bonferroni's *post hoc* correction (SPSS 18.0, IBM, USA) and the Student's *t*-test were used to evaluate statistical differences between different groups. Data were considered to be statistically significant at P<0.05.

## Results

### Identification of *Rab7*-knock-in mice

**Figure 1:**
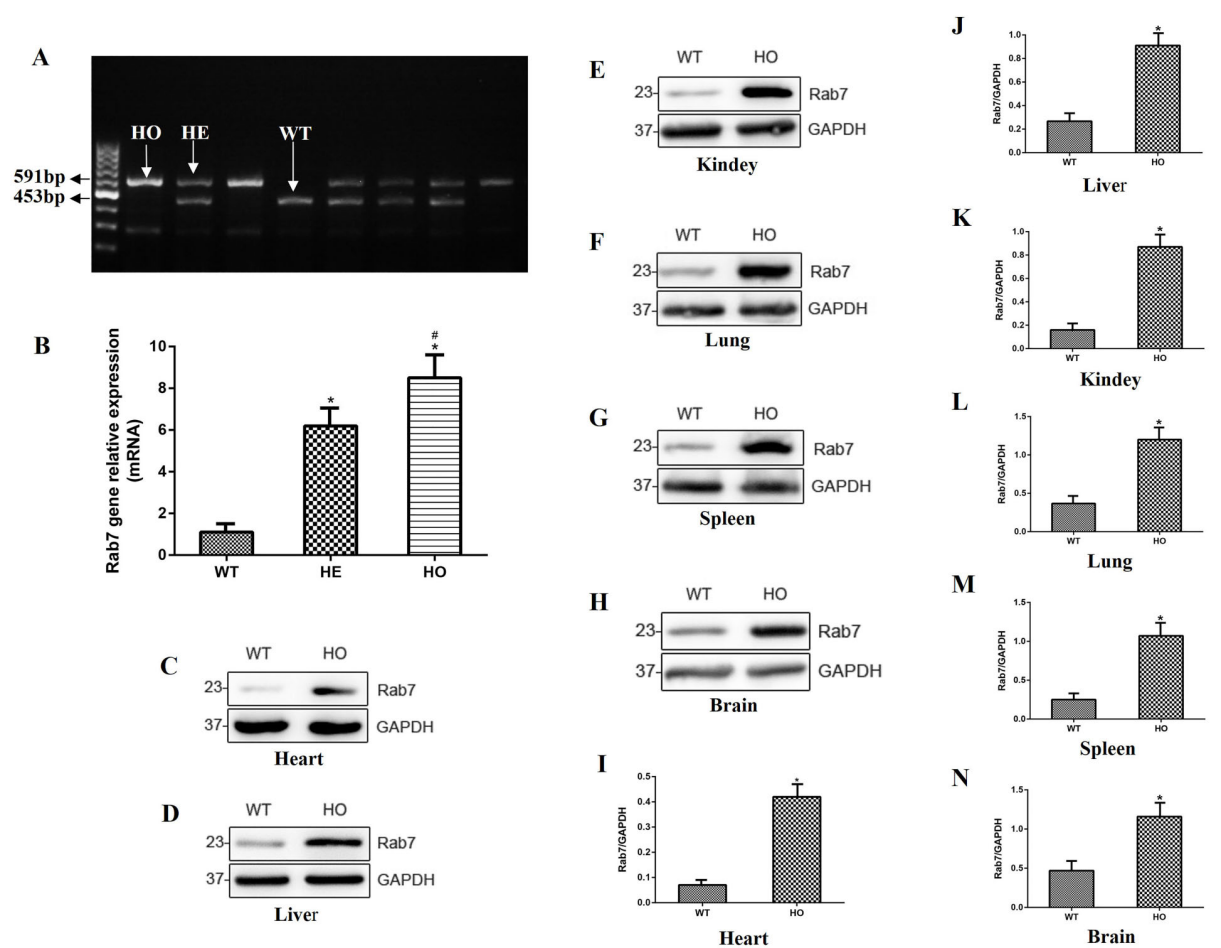
Identification of *Rab7*-knock-in mice was performed at the DNA, RNA, and protein levels. **A**, Results of agarose gel electrophoresis at the DNA level between the homozygous (HO) mice, heterozygous (HE) mice, and wild type (WT) mice. **B**, RNA levels of *Rab7* were assayed by qRT-PCR. *P<0.05, compared with the WT mice group. ^#^P<0.05, compared with the HE mice. **C**-**H**, Protein levels of *Rab7* were measured by western blotting in the heart, liver, kidneys, lungs, spleen, and brain. **I**-**N**, Relative protein levels of **C**-**H**. GAPDH was used as the internal standard. *P<0.05, compared with the WT mice group (ANOVA with Bonferroni's *post hoc* correction or Student's *t*-test).

To ensure that *Rab7* was highly expressed *in vivo* in the homozygous mice, identification was performed at the DNA, RNA, and protein levels. At the DNA level, different mouse genotypes were observed in each lane, showing a 591-bp band in the homozygous mice, a 591-bp band and a 453-bp band in the heterozygous mice, and only a 453-bp band in the WT mice (Figure 1A). At the RNA level, the expression of *Rab7* was highest in the homozygous mice and lowest in the WT mice (Figure 1B). Finally, the expression of *Rab7* in the heart, liver, kidneys, lungs, spleen, and brain was higher in the homozygous mice than in the WT mice (Figure 1C to N).

### *Rab7* expression in WT mice after UUO and autophagic activity determination

**Figure 2:**
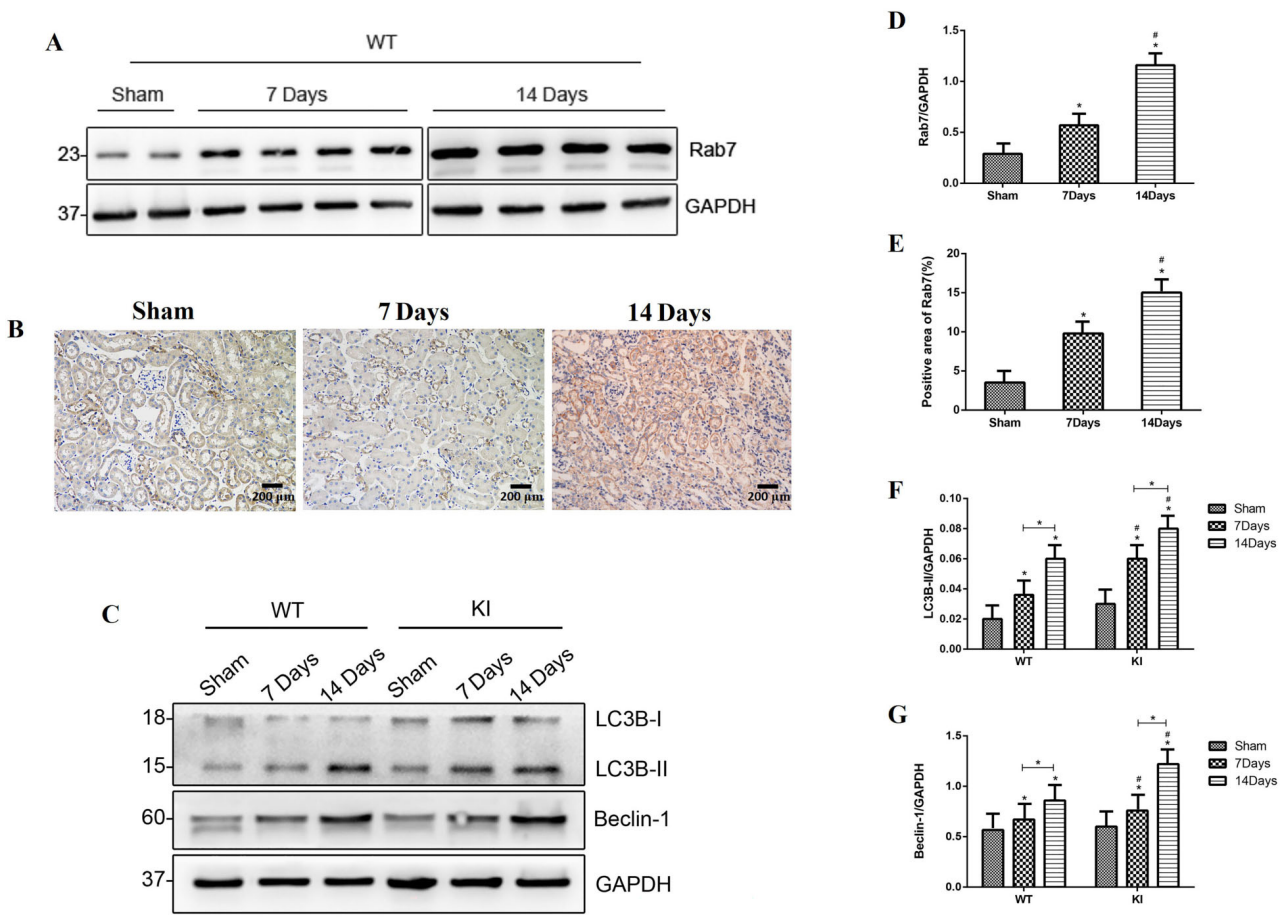
*Rab7* expression in wild type (WT) mice after unilateral ureteral obstruction (UUO) and autophagic activity determination in each group of mice. **A**, Protein level of *Rab7* in the WT mice after the UUO on day 7 and day 14. **B**, Immunohistochemical staining (200×, magnification bars 200 μm) of *Rab7*. **C**, Protein levels of LC3B-II and Beclin-1 in each group of mice. **D**, Relative protein level of **A**. *P<0.05, compared with the sham group. ^#^P<0.05, compared with the 7-day group. **E**, Positive area of **B**. *P<0.05, compared with the sham group. ^#^P<0.05, compared with the 7-day group. **F** and **G**, Relative protein levels of LC3B-II and Beclin-1. *P<0.05, compared with the sham group in WT and knock-in (KI) mice, and the 7-day group with the 14-day group in WT and KI mice. ^#^P<0.05, comparing the 7-day group in KI mice with the 7-day group in WT mice, and the 14-day group in KI mice with the 14-day group in WT mice (ANOVA with Bonferroni's *post hoc* correction).

*Rab7* expression did not significantly change in the sham-operation WT mice, 7 and 14 days after surgery. However, 14 days after the UUO, *Rab7* expression was higher than that after 7 days (Figure 2A and D) and immunohistochemical staining of *Rab7* showed the same result as western blotting (Figure 2B and E). Western blotting revealed that the expression of Beclin-1 and LC3B-II, which indicates autophagic activity, in WT mice was also higher 14 days after operation than 7 days after operation. Similarly, the autophagic activity in *Rab7*-knock-in mice was higher 14 days after operation than 7 days after operation. Notably, at both time points, *Rab7*-knock-in mice had higher autophagic activity than WT mice (Figure 2C, F, and G).

### Comparison of renal function between WT and *Rab7*-knock-in mice on day 7 and day 14

**Figure 3:**
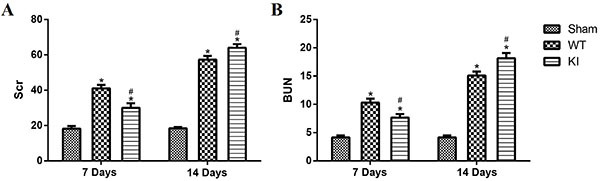
Renal function of *Rab7*-knock-in (KI) mice and wild type (WT) mice on day 7 and day 14. **A**, Serum creatinine (Scr, in μM) in each group. **B**, Urea nitrogen (BUN, in mM) in each group. *P<0.05, compared with the sham group on day 7 and day 14. ^#^P<0.05, comparing WT and KI mice on day 7 and day 14 (ANOVA with Bonferroni's *post hoc* correction).

On day 7 and day 14 after operation, no obvious abnormalities were observed in the sham-operation groups. On day 7, Scr and BUN of the *Rab7*-knock-in mice were significantly lower than those of the WT mice. However, on day 14, Scr and BUN of the *Rab7*-knock-in mice were higher than those of the WT mice (Figure 3A and B). Therefore, renal function of the *Rab7*-knock-in mice was better than the WT mice on day 7 and the results were reversed on day 14.

### Comparison of renal interstitial fibrosis between WT and *Rab7*-knock-in mice 7 days after UUO

**Figure 4:**
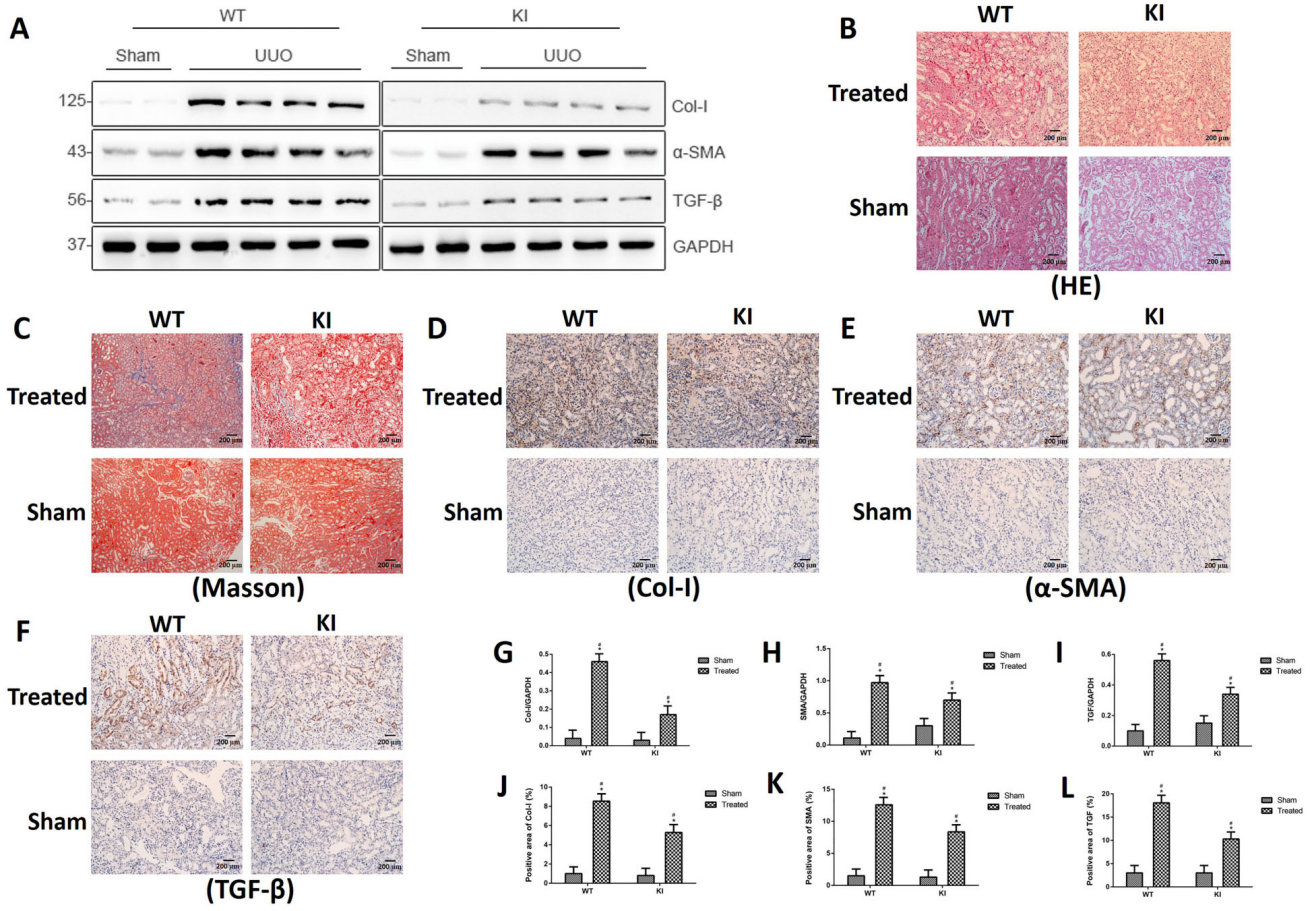
A, Protein levels of Col-I, α-SMA, and TGF-β at 7 days. **B** and **C**, Hematoxylin-eosin (HE) (200×, magnification bars 200 μm) and Masson (200×, magnification bars 200 μm) staining. **D**-**F**, Immunohistochemical staining (200×, magnification bars 200 μm) of Col-I, α-SMA, and TGF-β, respectively. **G**-**I**, Relative protein levels of Col-I, α-SMA, and TGF-β, respectively. **J**-**L**, Positive areas of Col-I, α-SMA, and TGF-β, respectively. *P<0.05, compared with the sham group in wild type (WT) or knock-in (KI) mice. ^#^P<0.05, comparing the treated group in WT with the treated group KI mice (ANOVA with Bonferroni's *post hoc* correction or Student's *t*-test).

Seven days after the operation, western blotting of protein expression in renal tissue showed that Col-I, α-SMA, and TGF-β expression was weak in the sham-operation groups (in both WT and *Rab7*-knock-in mice). In the two UUO groups, the expression of these three markers was weaker in the *Rab7*-knock-in mice than in the WT mice (Figure 4A, G-I). The kidneys of each group of mice were harvested for HE (Figure 4B) and Masson (Figure 4C) staining. There was no obvious pathological change in the kidneys of mice in the sham-operation groups (in both WT and *Rab7*-knock-in mice). In the two UUO groups, the renal tubules became atrophied and collagen fibers were deposited in the WT mice. In contrast, the renal structural damage and collagen deposition were relatively mild in the *Rab7*-knock-in mice. Immunohistochemical staining of Col-I (Figure 4D and J), α-SMA (Figure 4E and K), and TGF-β (Figure 4F and L) showed that their expressions were weak in the sham-operation groups (in both WT and *Rab7*-knock-in mice). In the two UUO groups, Col-I, α-SMA, and TGF-β expressions increased significantly in the WT mice and were stronger than those in *Rab7*-knock-in mice. Therefore, renal fibrosis was milder in the *Rab7*-knock-in mice than in the WT mice 7 days after UUO.

### Comparison of renal interstitial fibrosis between WT and *Rab7*-knock-in mice 14 days after UUO

**Figure 5:**
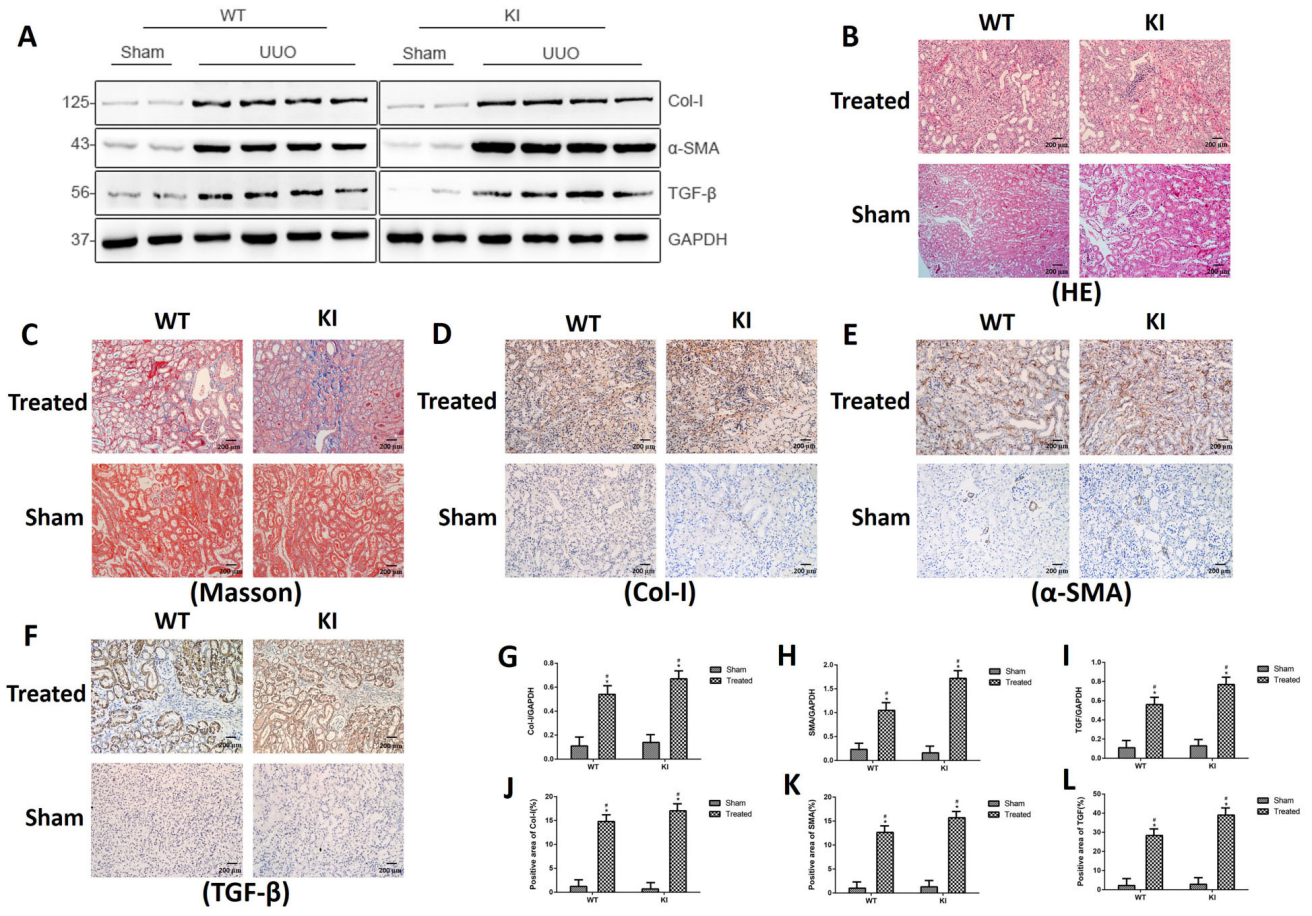
A, Protein levels of Col-I, α-SMA, and TGF-β at 14 days. **B** and **C**, Hematoxylin-eosin (HE) (200×, magnification bars 200 μm) and Masson (200×, magnification bars 200 μm) staining. **D**-**F**, Immunohistochemical staining (200×, magnification bars 200 μm) of Col-I, α-SMA, and TGF-β, respectively. **G**-**I**, Relative protein levels of Col-I, α-SMA, and TGF-β, respectively. **J**-**L**, Positive areas of Col-I, α-SMA, and TGF-β, respectively. *P<0.05, compared with the sham group in wild type (WT) or knock-in (KI) mice. ^#^P<0.05, comparing the treated group in WT mice with the treated group in KI mice (ANOVA with Bonferroni's *post hoc* correction or Student's *t*-test).

Fourteen days after surgery, western blotting of protein expression in renal tissue showed that Col-I, α-SMA, and TGF-β expression was low in the sham-operation groups (in both WT and *Rab7*-knock-in mice). In the two UUO groups, the expression of these three markers was stronger in the *Rab7*-knock-in mice than in the WT mice (Figure 5A, G-I). The kidneys of each group of mice were harvested for HE (Figure 5B) and Masson (Figure 5C) staining. There was no obvious pathological change in the kidneys of mice from the sham-operation groups (in both WT mice and *Rab7*-knock-in mice). In the two UUO groups, the renal tubules were atrophied and collagen fibers were deposited in the WT mice. In contrast, the renal structural damage and collagen deposition were more severe in the *Rab7*-knock-in mice. Immunohistochemical staining of Col-I (Figure 5D and J), α-SMA (Figure 5E and K), and TGF-β (Figure 5F and L) showed that their expressions were weaker in the sham-operation groups (in both WT and *Rab7*-knock-in mice). In the two UUO groups, the *Rab7*-knock-in mice had stronger α-SMA and TGF-β expressions than the WT mice. Therefore, renal fibrosis was more severe in the *Rab7*-knock-in mice than in the WT mice 14 days after UUO.

## Discussion

Renal fibrosis has complicated mechanisms. Previous studies suggest that it may be associated with multiple channels and regulated by multiple signaling pathways (23,24). Although autophagy has been found to be associated with renal fibrosis, it is unclear whether autophagy promotes or inhibits renal fibrosis with the upregulated autophagic activity. *Rab7* has been reported to be involved in the formation and transport of autophagosomes, and their fusion with lysosomes (3). However, whether *Rab7* plays a role in renal fibrosis remains undetermined.

In the current study, we used the latest CRISPR/Cas9 gene editing technology to establish *Rab7* transgenic mice. After passage and identification, only the homozygous mice were selected for the experiment as they could serve as ideal experimental animals for studying the relationship between *Rab7* and renal interstitial fibrosis. The renal fibrosis model prepared by UUO simulates the pathological changes seen in chronic kidney disease and has been recognized as an ideal model for research on renal fibrosis. We detected *Rab7* expression in the kidneys of WT mice 7 and 14 days after UUO. The results showed that *Rab7* expression increased over time, suggesting a certain relationship between *Rab7* and renal fibrosis. However, it is still unclear whether the increased *Rab7* expression is a protective mechanism of the body to suppress fibrosis in the kidneys or whether *Rab7* itself is a marker of the severity of fibrosis (i.e., the expression of *Rab7* increases with the worsening of fibrosis). Therefore, we compared renal function and the degree of renal fibrosis in WT mice and *Rab7*-knock-in mice on day 7 and day 14.

Seven days after UUO, renal fibrosis was milder in *Rab7*-knock-in mice than in WT mice and renal function was better. In addition, the autophagic activity was higher in the former. While it has been reported that the continuous activation of autophagic activity can cause damage to the kidney, the lower degree of renal fibrosis in the *Rab7*-knock-in mice in our study suggested that *Rab7* had functions other than suppressing renal fibrosis. Studies have demonstrated the close relationships of Rab7 with lipophagy and endocytosis (25,26). Rab7 plays a key role in regulating lipophagy as it promotes the phagocytosis of lipid droplets by autophagosomes. In addition, Rab7 participates in the endocytosis process of cells. Endocytosis is characterized by the transformation from early endosomes to late endosomes. Rab5 is present in the early endosomes. When early endosomes are transformed into late endosomes, the loss of Rab5 and the recruitment of Rab7 cause Rab7 to finally attach to the late endosomes. Therefore, the process of cell endocytosis can be regarded as the transformation between Rab5 and Rab7 (3,27). The milder fibrosis in *Rab7*-knock-in mice 7 days after UUO may be explained by the possible lipophagy and endocytosis activity of Rab7, during which some excessively deposited extracellular matrix and inflammatory cells are engulfed and degraded, thus delaying the progression of renal fibrosis in mice. In addition, studies have shown that in the early stage of chronic kidney disease, MMP-2 activity is increased, which can degrade Col-IV in the basement membrane, change the microenvironment of renal tubular epithelial cells, promote epithelial mesenchymal transition, and increase the production of extracellular matrix. MMP-2 also can activate the profibrotic factors, such as TGF-β1 and IGF-1. Previously, our group found that up-regulation of Rab's expression inhibited MMP-2 activity (28). Therefore, 7 days after UUO, the mild degree of fibrosis in *Rab7*-knock-in mice is likely to be related to the inhibition of MMP-2 activity.

The autophagic activity of the two groups increased with the progression of renal fibrosis. Fourteen days after UUO, *Rab7*-knock-in mice had stronger autophagic activity than the WT mice, but with more severe renal fibrosis and worse renal function. Studies have shown that autophagy is cell-specific in fibrogenesis. For example, autophagy induced in hepatocytes or hepatic macrophages can inhibit fibrosis (29), whereas autophagy induced in hepatic stellate cells can aggravate fibrosis (30). Furthermore, different autophagy-inducing methods can result in different body responses. On one hand, it is necessary to induce autophagy to degrade lipids during hunger and undernourishment, to provide energy for the whole body and maintain normal life activities (31). On the other hand, UUO-induced autophagy leads to excessive lipid deposition, and this lipid toxicity aggravates renal fibrosis (17). In our current study, the continuous activation of autophagic activity in *Rab7*-knock-in mice led to lipid deposition in the kidneys, causing severer renal fibrosis. It is estimated that *Rab7*-knock-in mice have stronger inhibition of MMP-2 activity than WT mice, and degradation of extracellular matrix is decreased, which is a possible cause of the eventually more serious fibrosis in transgenic mice.

Renal fibrosis is a necessary step during the continuous progression of primary or secondary kidney disease into renal failure, and effective measures against renal fibrosis can delay the progression of chronic renal failure. In the future, *Rab7* may be a therapeutic target for renal fibrosis, if the balance between the expression of *Rab7* and autophagic activity and the expression of MMP-2 can be found in the course of the disease. For this purpose, more laboratory and clinical studies are warranted.
